# A Reaction Time Experiment on Adult Attachment: The Development of a Measure for Neurophysiological Settings

**DOI:** 10.3389/fnhum.2016.00548

**Published:** 2016-11-02

**Authors:** Theresia Wichmann, Anna Buchheim, Hans Menning, Ingmar Schenk, Carol George, Dan Pokorny

**Affiliations:** ^1^Institute for Psychology, University of InnsbruckInnsbruck, Austria; ^2^Forel-Clinic, EllikonZürich, Switzerland; ^3^Rehaklinik BellikonZürich, Switzerland; ^4^Department of Psychology, Mills CollegeOakland, CA, USA; ^5^Department of Psychosomatic Medicine and Psychotherapy, Ulm UniversityUlm, Germany

**Keywords:** adult attachment projective picture system, reaction times, decision task

## Abstract

In the last few decades, there has been an increase of experimental research on automatic unconscious processes concerning the evaluation of the self and others. Previous research investigated implicit aspects of romantic attachment using self-report measures as explicit instruments for assessing attachment style. There is a lack of experimental procedures feasible for neurobiological settings. We developed a reaction time (RT) experiment using a narrative attachment measure with an implicit nature and were interested to capture automatic processes, when the individuals’ attachment system is activated. We aimed to combine attachment methodology with knowledge from implicit measures by using a decision RT paradigm. This should serve as a means to capture implicit aspects of attachment. This experiment evaluated participants’ response to prototypic attachment sentences in association with their own attachment classification, measured with the Adult Attachment Projective Picture System (AAP). First the AAP was administered as the standardized interview procedure to 30 healthy participants, which were classified into a secure or insecure group. In the following experimental session, both experimenter and participants were blind with respect to classifications. One hundred twenty eight prototypically secure or insecure sentences related to the eight pictures of the AAP were presented to the participants. Their response and RTs were recorded. Based on the response (accept, reject) a continuous security scale was defined. Both the AAP classification and security scale were related to the RTs. Differentiated study hypotheses were confirmed for insecure sentences, which were accepted faster by participants from the insecure attachment group (or with lower security scale), and rejected faster by participants from secure attachment group (or with higher security scale). The elaborating unconscious processes were more activated by insecure sentences with potential attachment conflicts. The introduced paradigm is able to contribute to an experimental approach in attachment research. The RT analysis with the narrative procedure might be of interest for a broader variety of questions in experimental and neurophysiological settings to capture unconscious processes in association with internal working models of attachment. An electrophysiological model based on preliminary research is proposed for assessing the preconscious neuronal network related to secure or insecure attachment representations.

## Introduction

Attachment theory is an evolutionary-based theory of a specific type of intimate human social relationship conceived to have a major developmental influence from “the cradle to the grave” (Bowlby, 1969, 1973). According to attachment theory, the foundation of the attachment relationship is a biologically based behavioral system that evolved in ways that influence and organize motivational, cognitive, emotional and memory processes. These processes are organized in early infancy with respect to significant caregiving figures that extend into adulthood. Bowlby (1980) conceived attachment as a key mechanism related to maintaining biological homeostasis, including the modulation of physiological stress and mental health. Researchers have found physiological correlates of attachment and the affective components of relationships in nonhuman species and humans. Mental representations of early attachment relationships shape emotional and cognitive information, which affects our attention and memory. In order to maintain organization within the attachment system, emotional reactivity is then regulated within the central nervous system (Bretherton, 1993; Main, 1995). Over the decades, psychobiological attachment research with infants and adults has increased dramatically (Coan, 2008; Gander and Buchheim, 2015). Attachment patterns have been linked to different ways to emotion regulation processes and some researchers even argued that the attachment system is in itself an emotion regulation device (Vrtička and Vuilleumier, 2012).

Most recent findings on attachment and neurobiology in functional magnetic neuroimaging (fMRI) showed that researchers investigated very different systems, often by very different means and a variety of paradigms, ranging from the presentation of individual photos of loved and unknown faces to more complex approaches (e.g., reflecting on attachment-relevant events, priming experiments, talking about attachment-relevant situations; see overview Buchheim and George, 2012). At present, the delineation of a neuronal network of attachment is not possible yet. However, there is evidence across fMRI studies that brain regions like the amygdala and orbito/prefrontal cortices are involved in processing attachment-related stimuli. In addition, convergent research results suggest that when caregiving is addressed, dopamine-associated regions of the reward system are active that differ from the neural correlates of the postulated “attachment circuitry” (Buchheim et al., 2010).

Several neurophysiological studies of adult attachment assessing the autonomic nervous system, the hypothalamic-pituitary-adrenocortical axis or frontal electroencephalography (EEG) asymmetry used self-report measures (Carpenter and Kirkpatrick, 1996; Kim, 2006; Laurent and Powers, 2007; Rochman et al., 2008; Zhang et al., 2008; Kiss et al., 2011; Dan and Raz, 2012), while other studies used narrative interview measures of attachment such as the Adult Attachment Interview (AAI) and the Adult Attachment Projective Picture System (AAP; Beijersbergen et al., 2008; Buchheim et al., 2009; Fraedrich et al., 2010; Holland and Roisman, 2010; Behrens et al., 2011; Leyh et al., 2016).

The self-report questionnaire instruments are conceived as personality constructs and assess the subjective evaluation of attachment styles with reported patterns monitored by conscious processing of feelings and experiences related to desires and worries regarding a romantic partner; these measures typically differentiate secure from insecure avoidant or anxious attachment styles (Ravitz et al., 2010). By contrast, developmental attachment measures such as the AAI (George et al., 1985–1996; unpublished manuscript) or AAP (George et al., 1999) are designed to activate the individuals attachment system by introducing attachment-related topics (e.g., separation, illness, abuse and death), and assess attachment representations (secure, insecure-dismissing, insecure-preoccupied and unresolved trauma) based on the analysis of discourse patterns of verbatim transcripts. Interview discourse analysis is less concerned with a specified response (as compared with attachment style measures) as how experiences and feelings are described.

In a very recent fMRI study, Yaseen et al. (2016) investigated the comparison of brain activity correlating with self-report (Relationship Scales Questionnaire, RSQ) vs. a narrative attachment measure (AAI) during conscious appraisal of an attachment figure. Interestingly the two measures elicited different brain responses. While the AAI appeared to disproportionately correlate with conscious appraisal associated activity in Default Mode Network (DMN) and *subcortical* structures, the RSQ seemed to tap Executive Frontal Network (EFN) structures more extensively. The authors suggested, that the AAI captured more interoceptive, “core-self”-related processes, while the RSQ assessed higher-order cognitions involved in attachment. The authors recommended in their conclusions, that the AAP might be an appropriate alternative in this kind of research, since this measure consists of a set of pictures feasible to present during an experimental setting.

The feasibility of the AAP measure as an attachment-activating stimulus in a neurobiological context (fMRI, neuroendocrinology) has been established in diverse experimental settings in clinical and nonclinical groups (e.g., Buchheim et al., 2006, 2008, 2009, 2012). Participants in these different settings were instructed to tell stories to the AAP picture stimuli in the fMRI environment (Buchheim et al., 2006, 2008) or were presented individualized sentences constructed from their own AAP responses in the fMRI setting (Buchheim et al., 2012).

In the context of a double-blind study with a neuroendocrine research question, we modified the AAP task for a double blind controlled study comparing the effects of oxytocin to a placebo condition. The AAP picture presentations were accompanied by prototypical phrases constructed to represent one of the four established attachment categories (i.e., a generalized attachment-sentence schema for each attachment group). The participants were instructed to rank these phrases from the most to the least appropriate for each presentation. The most interesting finding from this study was that insecurely attached individuals at baseline decided that secure attachment sentences were most appropriate for them under the oxytocin condition (Buchheim et al., 2009). This attachment experiment was a first attempt to assess a combination of conscious and unconscious processes in a self-report perception task. In this present study, we sought to improve on this approach by using this methodology in a reaction time (RT) paradigm. The research question was to examine if the RTs differed with respect to an individual’s attachment representation in order to provide a paradigm to use in a neurobiological setting, like an EEG experiment.

One interesting development in the past few years has been experimental research using the Implicit Association Task (IAT), the goal of which was to explore the domain of automatic cognitive processes concerning the evaluation of the self and others (Lane et al., 2007). The IAT task is based on the measurement of RTs and answers in combination with a target category, for example gender stereotyping and the self. Implicit measures have been successful in predicting verbal behavior, group membership, sexual behavior, and evaluative judgments (Gawronski, 2002) or personality (Grumm and von Collani, 2007).

RT research has a long experimental tradition in psychology, beginning with the experiments by Helmholtz (1850). Helmholtz was interested in the time relations structured by the nervous systems of living beings not just from a physiological but also from a psychological point of view. In fact, at the time at which he performed his time experiments in frogs, Helmholtz carried out similar studies in human beings (Schmidgen, 2002).

RT experiments are relatively inexpensive to execute and results are easy to obtain, even though the conclusive interpretations are still under discussion. According to Harris et al. (2014) RT experiments have become a standard paradigm for measuring behavioral reactions without taking into account underlying mental processes. Harris et al. (2014) suggested a sophisticated way to improve the analysis and interpretations of RT paradigms.

The idea behind the measurement of RT is that it can be used as a measure in social cognition research, as an index of the complexity of the underlying mental processes. Results showed for example, that more complex processes are associated with longer elaboration/RTs (Rösler, 1993). Moreover, RT experiments have a predictive value for social decisions and have been used successfully in IAT clinical and social experiments (Lane et al., 2007). It was possible to differentiate groups with and without disorders using the IAT in reliable experiments about self-judgments (Gemar et al., 2001). RT measures were also used to understand semantic priming. Reactions were more quickly facilitated when categories were closely related and shared the same reaction as compared to categories that did not share the same reaction. Attribution measures can be interpreted as a measure of relative identity with the objects (Lane et al., 2007). The association of the self as a target category and an attitude dimension provides a measure of implicit self-esteem; it describes the strength between associations of the self and another category. Studies show that emotional relevant primes have an effect on memory performance. One study showed that memory performance was impaired in borderline patients when negatively valued interference was presented (Mensebach et al., 2009). A recent study on autobiographical memories reported that past intentions could be reliably identified with high accuracy using a RT measure (Zangrossi et al., 2015).

A central concept of attachment theory is that individuals develop internal working models, that include expectations about the self, and significant others outside of conscious awareness (Bowlby, 1969, 1973, 1980; Bretherton, 1985). Furthermore, internal working model content is believed to include knowledge about concrete details of interpersonal experiences as well as the associated affect (Bretherton, 1985). In general, psychoanalytic theory suggests to divide the mind into three different levels: the conscious mind includes everything we are aware of and represents our mental processing that we think of and talk about rationally. A part of this includes memory structures, which are considered not always to be part of consciousness, but can be retrieved and brought into awareness, called preconscious. The unconscious mind constitutes a reservoir of feelings, thoughts, urges and memories that exist outside of conscious awareness. From a psychoanalytic point of view, most of these contents are unacceptable or unpleasant and represent feelings of pain, anxiety or individual conflicts. Unconscious processes are considered to influence our behavior and experience, even though we are unaware of these underlying influences (Freud, 1915/2001). As mentioned above internal working models are also thought to work primarily outside of conscious awareness (unconsciously) and guide attention, interpretation, and memory of attachment experiences and emotions. This allows individuals to generate expectations about the future concerning interpersonal interactions and to develop plans relating to them (Bowlby, 1969, 1973, 1980; Bretherton, 1985, 1990). Bowlby (1980) examined possible memory constructs and unconscious processes to explain misrepresentations of mental functioning and behavior, informed by mid-20th century advances in cognitive psychology (Bowlby, 1980). He suggested conscious representations of what parents made the child believe are stored in the semantic memory, while the defensively excluded and traumatic attachment experiences are stored in the episodic memory. Emotional schemata are part of episodic memories and, over time, these schemata can grow into explicit models of the self and the attachment figure (Liotti, 1999). According to information processing theory, the term “unconscious” describes the product of the perceptual systems that work unattended or unrehearsed. Thus from this perspective, nonconscious mental life is identified with early preattentive perceptual processes such as e.g., pattern or face recognition. One of the most common forms of preconscious processing is priming. When investigating the label “automatic”, some processes are intended, others require recent conscious and intentional processing of related information (Bargh et al., 2012). In the following, we use the term “unconscious” in association with the internal working models of attachment and “preconscious” when relating to information processing theory or neurobiological models.

There are several recent studies investigating implicit aspects of romantic attachment using self-report measures as explicit instruments for assessing attachment style (Marks and Vicary, 2015; De Carli et al., 2016). In the present study, we were interested to capture automatic processes in the moment the attachment system is activated by using a narrative attachment measure with an implicit nature. The AAP is designed to activate the individual’s attachment system and emphasizes the evaluation of unconscious defensive processes in the narratives. In this study, we intended to combine attachment methodology with knowledge from implicit measures by using a RT paradigm.

The general question for this study addressed how a person accepts or rejects prototypic sentences belonging to the two major attachment categories (secure and insecure) using a modified version of the AAP (Buchheim et al., 2009) in a RT paradigm. All participants were administered the standard AAP interview before the experiments started in order to assess their individual attachment representation. The participants did not get any information about their attachment representation during the whole assessments. The experimental design is described in the “Materials and Methods” Section in detail. In short, participants were presented pictures from the AAP accompanied with sentences representing different attachment patterns while assessing how long it took for them to make a decision (i.e., accept or not accept).

(1) We expected that participants would accept the prototypic sentences from the experiment more frequently when these sentences match with their own adult attachment classification.
(2) We expected group differences in reaction speed between participants with secure or insecure adult attachment classification assessed in the previous AAP interview. These expectations were differentiated for four possible configurations of the stimulus (secure, insecure) and the reaction (acceptance, rejection). Comparing both groups, we expected that
  (2a) secure sentences will be accepted faster by securely attached participants,
  (2b) secure sentences will be rejected faster by insecurely attached participants,
  (2c) insecure sentences will be accepted faster by insecurely attached participants,
  (2d) insecure sentences will be rejected faster by securely attached participants.
(3) The preference of secure or insecure prototypes in the experimental procedure was expressed by the continuous Adult Attachment Projective Relationship Choices Version 2 (AAP-RC) security scale (see below). We expected following correlations of the security scale with the reaction speed: the higher the security scale, …
  (3a) … the faster the acceptance of secure sentences,
  (3b) … the slower the rejection of secure sentences,
  (3c) … the slower the acceptance of insecure sentences,
  (3d) … the faster the rejection of insecure sentences.

## Materials and Methods

### Adult Attachment Projective Picture System (AAP)

The AAP (George and West, 2012) assesses the attachment status in adults using a standardized set of eight picture stimuli. The stimuli are line drawings that include a warm-up scene and seven attachment scenes of individuals in conceptually-defined attachment situations. Four so called “alone pictures” depict scenes of a single person with no other persons visible in the picture. Three so called “dyadic pictures” depict scenes of two or more persons in a potential attachment dyad. The scenes portray characters in different age groups across the life span (e.g., child to old age). The drawings contain only as much details necessary to connote the situation. Features indicating details such as emotion, ethnicity and gender are obscure. Stimulus presentation is standardized so as to introduce increasingly distressing attachment scenes. Participants are asked to tell a story to each picture using a standardized set of instructions: “What is going on in the picture, what led up to this scene, what are the characters thinking or feeling, and what might happen next.” AAP administration is done in person on an individual basis in a quiet location with no distraction and typically takes 30 min. The stories are audio-recorded and analyses are done from verbatim transcripts.

Each stimulus response is coded for attachment related content and defensive processes. Content coding evaluates representation of the presence and degree of integration (as defined by attachment research) of attachment relationships in the response, the actual coding dimensions of which are evaluated on different dimensions for the alone and dyadic pictures. Alone response content is evaluated on two dimensions. The agency of self is defined as the degree to which the character can seek and effectively use attachment figures. The connectedness is defined as the degree to which the character is portrayed as seeking proximity to others. Dyadic response content is evaluated for synchrony, a single dimension that captures the quality of agency of self and connectedness used for the alone pictures. Synchrony is defined as the degree to which responses depict attachment figure sensitivity in the context of distress themes (e.g., a child is sick) or mutual enjoyment in the context of togetherness themes (e.g., couple goes on a trip). Defensive processes are coded for the three standard attachment-defined defenses (Solomon et al., 1995): deactivation (distanced attention from attachment), cognitive disconnection (close attention to and confusion by attachment) and segregated systems (attachment fear and dysregulation).

The AAP designates four attachment classifications based on the evaluation of response content and defensive processes coding patterns across the entire set of seven attachment stories. Individuals are judged secure (F) when the coding patterns demonstrate that attachment figures are present and self and attachment figures manifest integrated interaction (sensitivity, relationship repair, thoughtful action and mutual enjoyment). Defensive processes, which can be depicted in any of the three defense, help integrate and maintain relationships, and manage attachment fears. Individuals with insecure-dismissing (Ds) or insecure-preoccupied (E) attachment are characterized by relative absence of integration, sensitivity and mutual enjoyment in their responses. Descriptions of the alone characters range from themes that portray taking simple action (reactive problem solving behavior without thoughtful consideration) to evidence, that characters cannot move forward. Attachment figures, if included, are described in ways ranging from functional roles without comfort (e.g., just give the sick boy soup), unable to respond (e.g., the mother refuses to hug the child), punishing and sometimes harsh (e.g., an enraged parent who is drunk and abusive). Connections with others, if described, are typically shift to interactions with non-attachment figures (e.g., police, nurse, soccer coach). The dismissing group is characterized by defensive processes, that deactivate attachment needs and shift attention away from attachment distress and themes (e.g., by rejection, power, achievement). The preoccupied group is characterized by defensive processes, that disconnect attachment needs and relationships (i.e., deconstruct the details) shifting attention to elements of frustration and anger, or distorting or blurring story characters and events (e.g., the child is waking up or going to bed; someone died—cannot specify who). Individuals are judged as insecure-unresolved with regard to trauma (U) when they remain dysregulated and overwhelmed by dangerous or threatening story elements (e.g., being frightened, assault, isolation, helplessness). One or more of their stories are void of the content and defensive processing features associated with integration, functional care, or attachment figure or other people providing care. For more complete details of the coding system and classification, see the monograph (George and West, 2012).

Multiple studies have demonstrated excellent convergent validity of the AAP with the AAI (George et al., 1985–1996), test-retest reliability, inter-judge reliability, and discriminant validity in community samples and clinical patients. Results from a large-scale psychometric investigation, including 144 adult participants demonstrated excellent inter-judge reliability; the concordance rate for two judges on the four-group classifications were 90%, *κ* = 0.85, test-retest reliability (after 3 months 84% remained in the same attachment category; *κ* = 0.78) and discriminant validity. To evaluate the convergent validity, AAP classifications were compared to independent AAI classifications. The concordance rates for the four-group classifications were 90%, *κ* = 0.84, and for the two groups (“secure” vs. “insecure”) even 97%, *κ* = 0.89 (George and West, 2001, 2012; Buchheim and George, 2011).

### Development of the AAP Reaction Time Paradigm

Buchheim et al. (2009) developed and used the first experimental adaption of the AAP in a double-blind, placebo-controlled within-subject experimental design. These researchers developed the AAP-RC stimulus set, which is comprised of a set of statements that represent attachment-related sentences that describe the AAP picture stimuli. The statements were schematic descriptions of secure, dismissing, preoccupied, and unresolved attachment stories, as determined by two expert AAP judges (AB, CG) who collectively had experience with over 300 AAP transcripts. Attachment statements described common story response situations. The study compared participant responses to the statement in an oxytocin and a placebo condition. The eight AAP picture stimuli were presented over four sequences, always presented in each sequence in the standardized order. Each of the 32 picture presentations was accompanied by four prototype phrases each of them representing one of the four established attachment categories. The participants were instructed to rank these phrases from the most to the least appropriate for each presentation. The phrases were presented in a randomized balanced sequence in order to minimize simple memory effects across test sessions.

**Figure 1:**
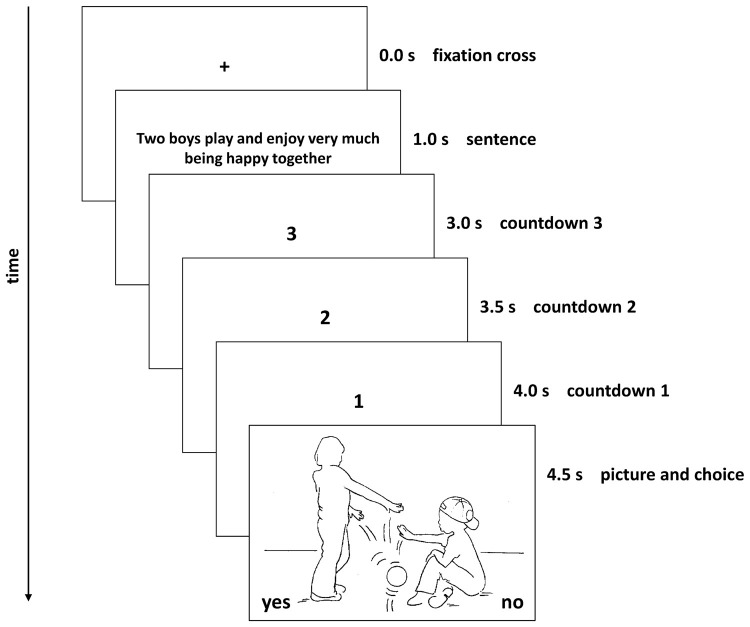
**One of the 128 screen sequences presented in the course of the experimental procedure**.

The present study used a modified version of the Buchheim et al. (2009) prototype sentences. Sentences were revised to improve the content and to control for the sentence length. In each group of length-adjusted sentences, all four sentences consisted of the same number of German words in order to minimize the effect of the sentence length on RTs. The modified system of 128 sentences is called Adult Attachment Projective Relationship Choices Version 2 (AAP-RC 2.0). The AAP-RC evaluation procedure uses all eight AAP drawings, including the first dyadic “warm-up” picture with two playing children. Hence, 64 sentences relate to the alone pictures and 64 sentences to the dyadic ones. The revised sentences were rated for content by three certified AAP judges. Table 1 shows example sets of four sentences that represent four attachment categories for two selected AAP picture stimuli. Figure 1 demonstrates an example how a stimulus sentence was presented on the PC screen to the participant. The experimental procedure consisted of 128 such screen sequences.

**Table 1:** **Examples of prototypical sentences from the Adult Attachment Projective Relationship Choices Version 2 (AAP-RC 2.0) instrument**.

Prototypical sentences from the AAP-RC	Attachment classification
**AAP picture “Bed”**
A child is put to bed by his mother and she sings a nice comforting lullaby for him.	**F**—secure
A child is put to bed by his mother and she gives him a toy and walks out.	**Ds**—dismissing
A child is put to bed by his mother and she is angry because he was too naughty.	**E**—preoccupied
A child is put to bed by his mother and she is helpless due to the child’s nightmare.	**U**—unresolved trauma
**AAP picture “Departure”**
A couple bids farewell and is looking forward to being together soon again.	**F**—secure
A couple bids farewell and he is ruminating about his upcoming business meeting.	**Ds**—dismissing
A couple bids farewell and she is very angry about his surprising departure.	**E**—preoccupied
A couple bids farewell and she threatens to hurt herself if he leaves her.	**U**—unresolved trauma

The RT paradigm used in this study followed Bem’s (1981) procedure for RT analysis for gender role schemas. The procedure was to record answers and to assess the time the participants needed to react. The Bem’s (1981) study showed that schema consistent judgments were more quick when the stimuli presented during a selection task matched participants’ gender role schema. We chose this approach because gender role schemas, like attachment representations, are conceived as stable views of self that develop in early childhood that automatically monitor, modulate attentional shifts and appraise new experiences (Bretherton, 1990). Classical experimental designs of self-concept tests using randomized stimulus sequences like the IAT (Gawronski, 2002) could not be used because attachment assessments such as the AAP must adhere to the procedural order in which stimuli are presented (George and West, 2012).

In the current study in the context of a diploma thesis (Wichmann, 2011, unpublished diploma thesis), we first administered the AAP and next presented the AAP-RC 2.0. We conducted an Attachment Reaction Time analysis (ART) for the experimental condition. The AAP was administered by a trained interviewer (TW). AAP verbatim protocols were coded by a certified AAP judge (AB). The structure of the administration procedure was as follows: the entire series of AAP picture stimuli were presented 16 times and each series was composed of eight pictures in the standardized AAP administration order. Each picture presentation was accompanied by one stimulus sentence, which was related to one of the four attachment representations. The experimental procedure contained a measure for the individual responses (yes/no) to the prototypical sentences and the recorded RTs.

The interview and the experimental task took place in the same office. The experimental condition was conducted using a computer. The computer was a table mounted Dell computer with no internet-connection and no additionally installed software. The program used for the presentation and RT measurement was *E-Prime* (Schneider et al., 2002). Participants were alone in the room. Room lighting was artificial and participants sat 0.5 m from the monitor. Answer responses were given via the computer keyboard. At the beginning of the experiment the participants were told to put the index fingers of their hands on the keys: Y for “yes” and the key N for “no”. The keys were marked with a red label. The participants had to press a key to move on with the task. A short practice task was given before to ensure that the participants had understood the task. The practice task included three attachment neutral stimuli with drawings produced in a style similar to the AAP pictures. All instructions were given on the computer screen and, if necessary, explained a second time after the test run.

Participants were told that the task was a speed task so as to avoid participant reflection and distraction. It was emphasized that there were no right or wrong answers, and that responses were simply their preferences. The timing of the presented sentence order (Figure 1) was as follows: 1st a fixation cross (1 s); 2nd the sentence (2 s); 3rd a countdown (1.5 s) and 4th the picture along with the decision task. AAP RC sentences were shown one at a time, next showing an AAP picture, with a “3-2-1” countdown shown between the sentence and the picture. The labels “Yes” and “No” were presented on the side of the monitor, analogs to the keys, during the decision tasks. The picture was displayed on the screen until a decision was made. The experiment continued only after a decision was made. We presented the participants first the sentence and then the picture so as to eliminate bias produced by different reading speed. The four attachment categories were presented each with four prototype sentences per picture. The order of attachment representations within the sentence was randomized. In sum there were 128 choices to be made. The choices were forced choice subjective selections, representing their acceptance (yes) or rejection (no) of an AAP RC sentence (see Table 2). Task scores are based on counts of agreements and rejections by four attachment representations.

**Table 2:** **Schema of evaluation of the AAP-RC 2.0 by a test person**.

Test person	F secure	Ds dismissing	E preoccupied	U unresolved trauma	Total
**Yes**	a	b	c	d	r
**No**	32 − a	32 − b	32 − c	32 − d	128 − r
**Total**	32	32	32	32	128

### Participants

Participants were asked for voluntary participation. The sample was comprised of 30 students from the University of Innsbruck (17 women, 13 men; sample mean age: 26.8, SD = 3.4). The participants reported no neurological conditions and were not in psychological or psychiatric treatment. All had normal or corrected eye vision. The study was conducted according to the Helsinki Declaration with informed consent received from all participants. All participants completed the study.

## Results

The reported results concern three methodical approaches: the AAP attachment classification assessment; the computerized experimental method AAP-RC; and the ART experiment. The results first describe the findings associated with each of the measures used in the study and second report the relations among them. There were no missing data.

### Adult Attachment Projective Picture System (AAP): Distribution of Attachment Classifications

The attachment classification distribution was as follows: 10 (33%) F, 12 (40%) Ds, 6 (20%) E, and 2 (7%) U. Because of the small frequencies in especially the preoccupied and unresolved groups, insecure classifications were collapsed together and data analyses compared only secure (*n* = 10, 33%) vs. insecure attachment (*n* = 20, 67%).

### Relationship Choices, Version 2.0 (AAP-RC): Psychometrical Analysis and Security Index

Reactions to AAP-RC stimuli in the ART test were coded dichotomously as yes (endorsement, acceptance) or no (rejection). The frequencies *a, b, c, d* shown in Table 2 represent numbers of accepted sequences belonging to the four attachment prototypes. The sets of 32 dichotomous items related to the attachment prototypes F, Ds, E, U, as well as the joint set of 96 insecure type items can be understood as a scale in the psychometric sense. These values of Cronbach α were satisfactory for the secure scale (0.77), for the U scale (0.82) for the joint insecure scale (0.88). However, they were *not* satisfactory for the Ds scale (0.64) and for the E scale (0.66). The correlation structure was investigated by means of the item-scale correlations and corrected item-scale correlations. The correlation structure was satisfactory for the system of two scales, secure and insecure. However, it was *not* satisfactory for the more detailed system of four scales F, Ds, E, U.

Guided by the referred psychometric results, we have decided to base the analyses of AAP-RC on the secure-insecure dichotomy. In respect of this aspect we have defined a *security index* expressing the degree of security vs. insecurity by the formula (see Table 2): *a/r* = *a/(a + b + c + d)*. The index is a proportion of accepted secure sentences related to all accepted sentences, ranging from 0.00 (completely insecure) to 1.00 (completely secure). By the random answering independent of sentence prototype, it would oscillate around 0.25. The analogously constructed complementary insecurity index *(b + c + d)/(a + b + c + d)* is mathematically redundant; summing up to one, both indices contain the same information. Hence, the following analyses utilize the *security index* as a central measure.

### Adult Attachment Reaction Time (ART): Reaction Time Analysis

The program *E-Prime* stored the dichotomous reaction and the needed RT in milliseconds. The hierarchically structured data sample consisted of 30 persons × 128 sentences = 3840 pairs of reactions and RTs.

**Figure 2:**
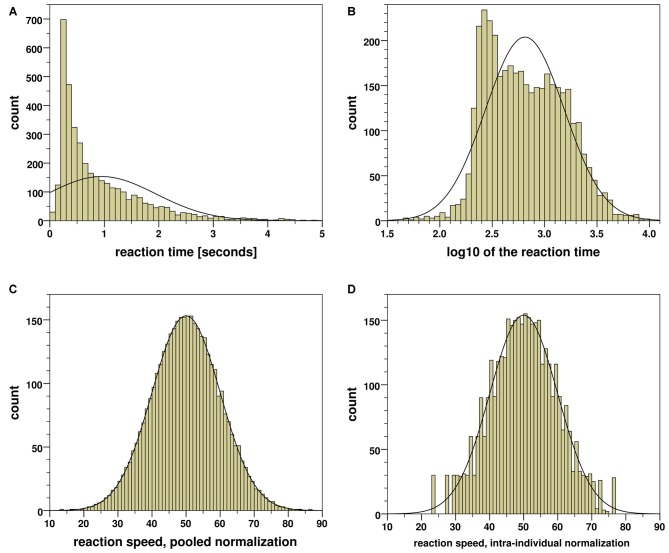
**Distribution of reaction time (RT), *N* = 3840 measurements. (A)** Original observation (range 0–15 s), **(B)** logarithmic transformation, **(C)** pooled normalization, **(D)** intra-individual normalization.

Figure 2A: the average RT was about 1 s, ranging from 0 up to 15 s; exact values of measures and statistics see in Table 3. As commonly experienced by the duration time data, the distribution was skewed and its normality was rejected by the Kolmogorov-Smirnov test.

**Table 3:** **Kolmogorov-Smirnov test of normality of the reaction time (RT) distribution**.

	Reaction time [ms]	log_10_(time[s])	Speed pooled normalization	Speed, intra-individual normalization
See Figure:	2A	2B	2C	2D
***N***	3840	3840	3840	3840
***M***	949	2.811	50.0000	50.0000
***SD***	999	0.376	9.9996	9.9507

**KS statistic^1^**	0.195	0.060	0.002	0.005
***p***	*p* < 0.001	*p* < 0.001	1.000	1.000

^1^*The largest absolute difference between empirical and theoretical distribution, exact two-tailed test*.

Figure 2B: this was the case after applying the frequently recommended logarithmic transformation. Additional problems were caused by some extreme outlier values. Similarly, other transformations considered by Harris et al. (2014) did not lead to satisfactory results in this case.

Figure 2C: transformation based on quantiles in the total pooled sample of 3840 measurements resulted in a close approximation to the normal distribution; the variable was transformed by the linear function *s*_(z)_ = 50–10*z*. The resulting variable is interpreted as the *speed* of the reaction. However, there were striking differences in the RTs between the 30 study participants, on an average ranging from 0.32 s up to 2.41 s. The reaction speed differed significantly by ANOVA (*F*_(29,3810)_ = 74.17, *p* < 0.001; *η*^2^ = 0.36); a considerable portion of measurement variance was explained by the individual basic reaction speed.

Figure 2D: in regard to excluding the bias by individual basic reaction speed, we have normalized speed values intra-individually. The RTs for a test person were replaced by ranks 1 for the slowest reaction to 128 for the quickest one, and transformed to the *s* (speed) values according to the quantiles of the normal distribution *N*(50,10)^1^. Because of the subsample sizes *n* = 128, the density curve of the obtained empirical distribution is less smooth than the previous one. Nevertheless, it is very close to the normal distribution *N*(50,10), and its normality in the sample of 3840 observations was *not* rejected by the exact Kolmogorov-Smirnov test.

The tests of study hypotheses (correlations, *t*-tests) were based on the last described intra-individually normalized speed values. The *N* = 3840 single values were aggregated to the intra-individual means for each of *N* = 30 study participants. Particularly, the following four aggregated values were relevant: speed of “yes” reactions to secure sentences; speed of “no” reactions to secure sentences; speed of “yes” reactions to insecure sentences; and, speed of “no” reactions to insecure sentences.

### Convergent Validity Between the AAP Interview and the AAP-RC Security Index

**Figure 3:**
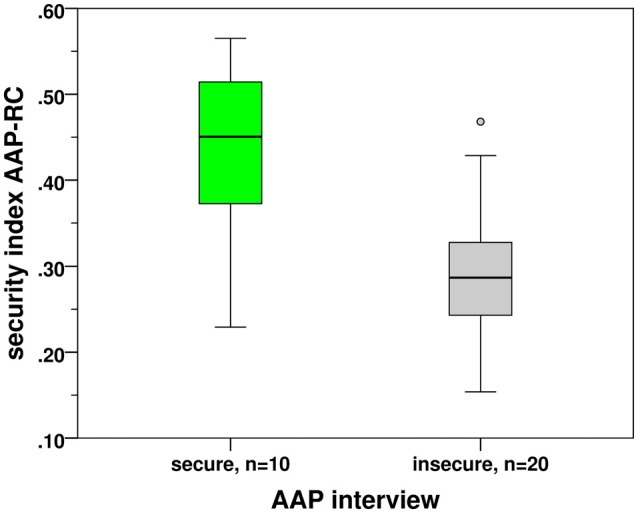
**Adult attachment projective relationship choices version 1 (AAP-RC) security index in attachment groups by AAP**.

The convergent validity of the AAP-RC security index was examined by its comparison with the secure and insecure attachment classifications (Figure 3). Mean of the AAP-RC security index in the secure group (*n* = 10, *M* = 0.432, SD = 0.105) was greater than the mean in the insecure group (*n* = 20, *M* = 0.293, SD = 0.087); this difference was significant according to the two-sided two-group *t*-test: *t*_(28)_ = 3.866, *p* = 0.001, Cohen’s *d* = 1.50 indicated a strong effect.

The predictability of attachment classifications on the basis of the security index in the attachment RT experiment was estimated by the discriminant analysis. The cross-validated classification was used, which is by small sample sizes particularly important (see “discriminant—cross-validation” in the IBM SPSS software system). The procedure recommended predicting AAP classification as secure when the security index exceeded the threshold 0.362 shown by horizontal line in Figure 3. Appling this threshold, 8 of 10 secure participants and 16 of 20 insecure participants were recognized correctly; the prediction was successful in 80% of cases in both groups.

### Reaction Time to Accept or Reject Secure or Insecure Prototype Sentences

For each participant, the set of 128 measurements was divided by sentence prototype stimulus (secure, insecure) and his/her answer reaction (yes, no) into 2 × 2 = 4 subsets, as described above (see “Adult Attachment Reaction Time (ART): Reaction Time Analysis” Section). Within each subset, the intra-individual mean values of speed were computed, resulting in the speed values of the following four stimulus-reaction combinations: (1) accept secure sentences; (2) reject secure sentences; (3) accept insecure sentences; and (4) reject insecure sentences. These computations were based (a) on all 128 sentences and alternatively; (b) on 64 sentences relating to the alone pictures; and (c) on 64 sentences relating to the dyadic pictures.

These speed variables were compared by ANOVAs for 2 × 2 repeated measures in the whole sample of *N* = 30 participants. Results of analyses based on all, alone and dyadic stimuli are shown in Table 4: (a) The analysis based on the complete material has shown that the *interaction of sentence prototype and answer* was significant (*p* = 0.011), whereas both main effects were not. As can be seen, participants answered more quickly to “yes to secure” and “no to insecure” and more slowly to “no to secure” and “yes to insecure”. It means that the “secure-conform” answers were given more quickly than “insecure-conform” ones. (b) For the alone pictures, none of the three ANOVA effects was significant. (c) For the dyadic pictures, the *interaction* effect (*p* = 0.005) and the main effect *sentence prototype* (*p* = 0.020) were significant. The highest speed was observed for the combination “yes to secure”; the lowest speed and hence the highest time needed to answer was observed for the combination “yes to insecure”.

**Table 4:** **Speed of yes/no answers to secure/insecure prototype sentences**.

	PROTOTYPE × ANSWER				ANOVA		
	Secure yes	Secure no	Insecure yes	Insecure no	Effect prototype	Effect answer	Effect interaction
	*M*	*M*	*M*	*M*	*F*_(1,29)_	*F*_(1,29)_	*F*_(1,29)_
	*(SD)*	*(SD)*	*(SD)*	*(SD)*	*p*	*p*	*p*
All stimuli	51.25	48.89	48.85	50.05	2.290	1.026	**7.320**
	(2.90)	(2.90)	(2.16)	(1.30)	0.141	0.319	**0.011**
Alone stimuli	49.53	48.89	49.71	50.29	1.661	0.002	0.641
	(4.17)	(4.90)	(3.55)	(1.81)	0.208	0.968	0.430
Dyadic stimuli^x^	52.14	49.40	47.50	49.81	**6.089**	0.278	**9.258**
	(3.83)	(5.71)	(2.91)	(1.84)	**0.020**	0.603	**0.005**

*Repeated measures in the sample of N = 30 participants. *Bold*: significant effects. ^x^sample n = 28, df = 1, 27*.

### Reaction Times in Secure and Insecure Attachment Groups According to AAP

The four speed variables (1–4) described in “Reaction Time to Accept or Reject Secure or Insecure Prototype Sentences” Section were compared between secure and insecure AAP attachment classification groups. As shown in Table 5, the significant group differences were found for “RC-insecure” prototype sentences:

1. The insecure participants *accepted* the RC-insecure sentences more quickly than the secure participants.
2. The secure participants *rejected* the RC-insecure sentences more quickly than insecure participants.

**Table 5:** **Speed of answers in participants with secure and insecure attachment according to the AAP classification**.

AAP-RC 2.0		AAP secure *n* = 10		AAP insecure *n* = 20		Cohen effect size		Two-group t-test	
Prototype	Answer	*M*	*(SD)*	*M*	*(SD)*	*d*	*t***_(28)_**	*p*
**All stimuli**
Secure	Yes	51.34	(2.81)	51.20	(3.01)	+0.05	+0.123	0.903
Secure	No	47.65	(5.90)	49.51	(3.08)	−0.44	−1.139	0.265
Insecure	Yes	47.00	(2.46)	*49.77	(1.24)	**−1.60**	**−4.134**	**0.000**
Insecure	No	*50.73	(0.86)	49.71	(1.37)	**+0.84**	**+2.164**	**0.039**
**Alone stimuli**
Secure	Yes	49.80	(3.11)	49.39	(4.68)	+0.10	+0.250	0.804
Secure	No	47.06	(5.45)	49.81	(4.46)	−0.57	−1.475	0.151
Insecure	Yes	47.70	(3.68)	*50.72	(3.11)	**−0.91**	**−2.359**	**0.026**
Insecure	No	50.56	(1.60)	50.16	(1.94)	+0.22	+0.565	0.576
**Dyadic stimuli**
Secure	Yes	52.03	(3.77)	52.19	(3.95)	−0.04	−0.108	0.915
Secure	No^x^	49.75	(7.01)	49.26	(5.3ß)	+0.08	+0.201	0.842
Insecure	Yes	45.91	(3.76)	*48.29	(2.07)	**−0.87**	**−2.256**	**0.032**
Insecure	No	*50.82	(1.47)	49.30	(1.82)	**+0.88**	**+2.277**	**0.031**

Group comparison. *Bold*: significant t-test. *significantly higher group mean. ^x^sample n = 28, AAP secure n = 8, df = 26.

The first result was also confirmed for both subsets of alone and dyadic sentences. The second result was confirmed for sentences connected to the dyadic pictures. In sum, the differences between the secure and the insecure attachment group according to the AAP were significantly manifest for the RC-insecure sentences.

### Reaction Times in Correlation to the Security Index AAP-RC in ART

**Figure 4:**
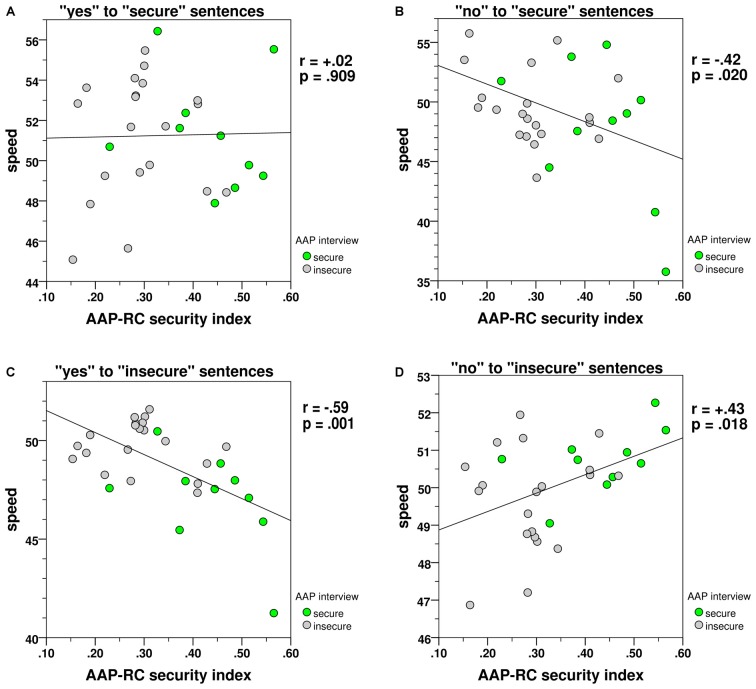
**Correlations between AAP-RC security-insecurity index and reaction speed. (A)** Accepted secure sentences, **(B)** rejected secure sentences, **(C)** accepted insecure sentences, **(D)** rejected insecure sentences.

The AAP-RC security index (see “Relationship Choices, version 2.0 (AAP-RC): Psychometrical Analysis and Security Index” Section) ranges from completely insecure (0.0) to completely secure (1.0) reactions to the 128 stimuli. The correlations of the AAP-RC security index with variables concerning the reaction speed by four stimulus-reaction pairings are shown in Figures 4A–D.

(A) The RT speed of acceptance of secure sentences (Figure 4A) was *not* significantly correlated with the security index; according to ART both rather securely or insecurely attached persons reacted comparable quick in this case.

(B) The rejection speed of secure sentences (Figure 4B) was negatively correlated with the security index; the rather insecurely attached persons rejected the secure sentences more quickly.

(C) The acceptance speed of insecure sentences (Figure 4C) was negatively correlated with the security index; the rather insecurely attached persons accepted the insecure sentences more quickly.

(D) The rejection speed of insecure sentences (Figure 4D) was positively correlated with the security index; the rather securely attached persons rejected the insecure sentences more quickly.

Summarizing, the results of the experiment—especially for insecure prototype sentences—indicate a consistency between the attachment preferences and the higher speed of the corresponding reaction. The complete results of the experiment are summarized visually in Figures 4A–D, which underlines the consistency of the different results.

## Discussion

### Discussion of the Methodology and Results

Bowlby (1980) proposed that inner working models of attachment function automatically and outside of conscious awareness. RT analyses are a way to observe the implicit automatic reactions. Pietromonaco and Barrett (2000) recommend the use of implicit measures like RTs to capture the unconscious parts of attachment, which are obscured when relying on self-report measures. Therefore we chose a narrative attachment measure, the AAP, designed to elicit unconscious elements by focusing on defensive processes, in combination with a RT measure. Our results reflect that implicit methodology can bring new and interesting insights in attachment related processes in the domain of neuroscience of human attachment.

Participants in our study were tested in an attachment RT experiment using the AAP picture stimuli accompanied by prototypic sentences representing different attachment representations (AAP-RC). Participants were not informed about their attachment classification prior to beginning the experimental session; therefore their reaction to the sentences was considered to be automatic and outside of conscious appraisal (i.e., unconscious). We hypothesized that the participants would accept the prototype sentences in the attachment RT paradigm: (1) more frequently; (2) more quickly when sentences matched with their own representations of attachment classification; and (3) that this would correspond with their attachment prototype preferences in the RT experiment (ART). Overall most of our expected results were confirmed for secure vs. insecure attachment groups.

The distribution of attachment classifications in our sample showed an overrepresentation of dismissing participants as compared to the distributions in samples with healthy controls (Bakermans-Kranenburg and van Ijzendoorn, 2009). Therefore, one caveat of our study is that we did not have a representative distribution of attachment classifications. Another caveat is the small sample size. The consequence was that data analyses for separate attachment groups was not possible and we were confined to comparisons of participants with secure and insecure attachment representations. This remains a challenge for our next studies.

Item-scale analyses confirmed the internal consistency for the secure and insecure scales. Discriminant analysis showed that AAP-AAP RC 2.0 convergence prediction was successful in 80% of the cases in both groups; 8 of 10 secure and 16 of 20 insecure participants. Although a 100% correspondence was not reached, there was a sufficient agreement in this study to demonstrate the validity of the paradigm. This association was stronger, for example, than the results of studies that correlated narrative and self-report attachment measures (e.g., Roisman, 2007).

Given that participants did not know their own attachment classifications by the standard AAP procedure, we can assume that they were not guided by informed conscious appraisals of attachment while evaluating the prototype sentences, rather by unconscious processes. The fact that the different measures showed a considerable convergence supports the conclusion that we were able to capture both conscious and unconscious automatic reactions to attachment related stimuli. The average RTs differed significantly between the study participants. The considerable portion of variance of the originally measured RTs is explained by the individual basic speed of reactions to the presented stimuli. This empirically found fact can be caused by different plausible reasons, like overall speed or slowness of mental processes of the subject, extended rational reasoning on the presented sentences, or intensive imagination triggered by them.

With regard to the RT results, we found that all participants had a tendency to answer “yes” to secure and “no” to insecure sentences quickly and more slowly when the cases were inverted (i.e., “no” to secure and “yes” to insecure). ANOVA did not show significant results for both main factors sentence prototype stimulus and answer reaction; the *interaction effect prototype and answer* was significant however. The participants accepted secure prototype sentences and rejected the insecure prototype sentences more quickly. One possible explanation of this finding is social desirability, because the perception of secure sentences could be expected to be ideal. This is in line with findings by De Carli et al. (2016). In their IAT study about caregiving and attachment, which they proposed as two different systems, the authors found that adult attachment style had a role in shaping the implicit attitude, but not the explicit attitude, concerning the category “mother.” The explicit attitude did not appear to be influenced in that study by experimental manipulation or the participants’ attachment style. The authors discussed that this can be explained by social desirability, because the perception of mother is expected to be mostly positive. In sum the IAT findings of De Carli et al. (2016) in the context of the transmission of attachment are in line with our results by showing that the participants preferred a particular style of caregiving coherent with their own attachment style. However the authors pointed out that their attachment measure was a self-report instrument that captured explicit thoughts only. However a notable strength in our study is that we used a free-response narrative attachment assessment measure, which seems to be more appropriate for this kind of experimental approach because of its implicit nature. Yet the role of social desirability should be clarified in future studies.

Despite the results, that all participants in our study accepted the prototype secure sentences faster than insecure prototypes, there were significant differences between the two adult attachment groups. Secure participants accepted more prototype secure sentences and showed faster RTs than insecure participants. Insecure participants accepted more insecure sentences, and did so faster than secure participants. This result underscores the presence of automatic unconscious detection and appraisal processes when responding to attachment relevant information.

Parallel patterns were found in the AAP-RC with the RTs in the experiment. Participants with higher preference for secure prototype secure sentences rejected insecure sentences more quickly. Participants with higher preference for insecure prototype sentences accepted insecure sentences more quickly and rejected secure ones more quickly.

Our differential hypotheses addressed secure and insecure prototype sentences. Findings supported our hypotheses, and we confirmed all hypotheses concerning the insecure prototypes. In other words: “*accept secure and reject insecure”* goes *fast*, and “*reject secure and accept insecure”* goes *slow*. It seems that the “insecure-type” reactions demand more time.

In a study by Rösler (1993), more complex processes took longer elaboration time than more simple ones. What makes the insecure reaction more demanding than the secure one in our study? We can nearly exclude that the linguistic or cognitive complexity would play a role: the grouped sentences had the same length and they were clear and understandable. However, the complexity of the relationship related decision processes might differ. It is reasonable then to conclude that the differences were due to story content. George and West (2012) described, how different insecure attachment representations are connected to different defensive mechanisms. The insecure attachment prototypes have the potential to address inner conflicts (e.g., ambivalence or deactivation of attachment relevant information), which must be recognized first, and then accepted or rejected. This unconscious process might request the additional “working time”^2^.

Our findings are in line with those of Vrtička et al.’s (2012) study of attachment style. These researchers used an explicit choice paradigm and found distinct effects of attachment avoidance and anxiety on subjective emotional judgments. Their results supported the assumption that anxious attachment is associated with a hyperactivating tendency for the appraisal of social threat, but may also involve an ambivalence influencing the judgment of information. Although, the authors did not use a RT experiment, their results support thinking that proposes that insecure attachment seems to need more mental elaboration time.

Therefore we could have assumed in our study that individuals with preoccupied attachment representations associated with heightened emotional reactivity would show different RT patterns compared to dismissing individuals, characterized by deactivating attachment related emotions. This important differentiation should be the next step in future studies with a larger sample size.

According to previous research with the AAP we might have also expected particularly differentiated results for the analyses based on “alone pictures” compared to “dyadic” ones. Alone pictures represent scenarios of emptiness and loneliness and seem to elicit high affective arousal in participants (Buchheim and George, 2011). However the results of the present study showed that insecure individuals needed longer elaboration times confronted with the dyadic pictures. This type of sentences (like in the AAP picture of the couple in the scenario “departure”) represents explicit attachment related scenarios between two or more persons (potential separation, need for care). We might conclude that insecure individuals needed more elaboration time for processing these attachment related conflicts. The observed differences should be verified in further investigations using a larger sample.

In sum, high security index scores were associated with prompt rejection of insecure prototype sentences. Lower security index scores were associated with prompt acceptance of insecure sentences, as well as rejection of secure sentences. Some other hypotheses could not be confirmed significantly; there were no contrary findings nevertheless. We might have demonstrated that the secure vs. insecure attachment classification groupings could be observed with the implicit measure, by observing the activation of inner working model in “real time.”

Our results support the conceptualization of inner working models of attachment as guiding attention and interpretation outside of conscious awareness and the coherency of the association between mental representation and interpretation of attachment situations (Bowlby, 1980).

From a methodological perspective, we suggest that the observation of RTs is valuable to complement the spectrum of mainstream measures in human neuroscience, like brain mapping or EEG analyses. These highly advanced measures focus on brain localizations and processes associated with different psychological tasks and events. The RT approach investigates the overall time of participants to specific stimuli analogous to the time complexity theory in computer science (Sedgewick and Wayne, 2011). The more operations are needed for the problem solution, the more time is needed. The time needed for the problem solution might then constitute an operationalization of the problem complexity and depends on numerous biasing factors. Human processing time consists of the individual’s basal or momentary reaction speed including external disturbing influences, which could cause long outlier RTs. The data-analytic procedure proposed in this article was designed with the aim to be robust against the mentioned biasing factors and could be a fruitful additional approach in an EEG analysis when using a similar paradigm.

### Limitations

The size of our sample of 30 participants was sufficient, albeit small, for the experimental investigation of the RT phenomena. The number of 128 attachment prototype sentences was considerably larger than the sample size; this circumstance limited the use of more advanced psychometrical analyses (e.g., factor analysis). Similarly, the sample sizes and the distribution of four particular attachment groups led us to the decision to confine the analyses to the two basic attachment classifications secure and insecure. In fact, secure vs. insecure analyses are often chosen as a comparison in the field of attachment. However as we discussed it would have been valuable to differentiate the insecure attachment groups and the individuals’ RTs. This aspect should be tested in further research.

In sum the present study served as a pilot study to test its feasibility in healthy participants. The next steps are the application of the RT experiment in clinical studies with a larger sample. Moreover, the AAP measure is constructed and validated for adults and adolescents only, so the application is limited to that age group and not feasible for children, where other measures should be used, like the Separation Anxiety Test (Klagsburn and Bowlby, 1976).

Despite these limitations, the study has shown that the concept of immediate reactions to stimulus sentence could be beneficial for experimental attachment research contributing to measure the intensity of unconscious processes empirically. As a following research step, we intend using psychometric procedures to continue and improve the development of the AAP-RC instrument in order to implement it in a neurobiological setting.

### Outlook: Neurobiological Model Using the Reaction Time Experiment on Adult Attachment

In the presented study, we have seen that stimuli with more distressing attachment content might need a longer RT for its elaboration than stimuli with more harmonious content. Future studies need to replicate these findings using larger samples. A further next step is to adapt the experiment for an EEG setting, which could give further insight into the neural mechanisms of potential response delays during an implicit task.

One of the most interesting areas in the research of preconscious perception is the investigation of early brain potentials. Until now, there are only a small number of studies examining the perception of emotional stimuli in individuals with different attachment patterns. In an EEG setting the N1 potential, which is also called N170 component, is considered to be a very sensitive representation of early perceptual processing. Spatio-temporal analyses of brain activity patterns during the first 200 ms after stimulus presentation have characterized the timing of attentional selection processes and different stages of feature encoding and pattern analyses (Hillyard et al., 1998). In an attachment study on *face recognition* Zhang et al. (2008) reported distinct differences in N1 activation using self-reports. The perception of angry faces was followed by high N1 amplitudes in anxious and secure individuals in contrast to the smaller amplitudes in avoidant individuals. Given that N1 is considered to be an index of the level of attention, the authors suggested that individuals with anxious attachment “use most, and avoidant individuals use least attentional resources to face stimuli than secure individuals”. The authors considered these differences as the results of automatic processes in association with conscious and preconscious emotional information processing. In contrast to the latter study Fraedrich et al. (2010) focused on event-related potentials (ERPs) in mothers during the perception of infant emotions by presenting positive, negative and neutral facial expressions as well as non-facial stimuli within an oddball paradigm. Dismissing mothers exhibited elevated N170 amplitudes for *facial* target stimuli within conditions that contained frequent non-facial stimuli. In summary, the findings suggested that insecure mothers require more cognitive resources to process infant faces, while secure mothers allocate more attention to infant faces and clearly show a perceptual bias toward social information. The differences between the study results of Zhang et al. (2008) and Fraedrich et al. (2010) might be due to the different stimulus material.

In a very recent study by Leyh et al. (2016), the association between maternal attachment representation and brain activity (ERPs) underlying the perception of infant emotions was examined. Securely attached mothers recognized emotions of infants more accurately than insecurely attached mothers. ERPs yielded amplified N170 amplitudes for insecure mothers when focusing on negative infant emotions. Secure mothers showed enlarged P3 amplitudes to target emotion expressions of infants compared to insecure mothers, especially within conditions with frequent negative infant emotions. In these conditions, P3 latencies were prolonged in insecure mothers.

One potential limitation of attachment research of preconscious perception with the help of the early brain potentials so far might be the predominant focus on *face processing* as the stimulus material. Neural processing in secure and insecure subjects were not examined by attachment related material directly linked to the individuals’ own attachment representations using a paradigm where spontaneous preferences had to be given in a defined time frame.

In a recently published article by Matheus-Roth et al. (2016) early occipital ERP’s (e.g., P100 and N170) have been shown to be sensitive for a “preference” for stimuli with alcohol association in patients with alcohol dependance. The authors used a Go-NoGo paradigm with three visual stimuli: tea, juice and beer. The N170 amplitudes were elevated in response to the alcohol-related (beer) stimuli in the NoGo condition in these patients compared to controls. The patients had to react to the frequent tea stimuli and ignore the beer and the juice stimuli. While the higher N170 component correlated with a relapse within the following 3-month, the shorter P100 latencies were related to higher depression scores. The latencies of these early ERPs represent the “RTs” of the brain, presumably independent of deliberate influence. In another study, the so called “mismatch negativity” (MMN) has been demonstrated to react pre-attentively to syntactic or semantic errors (Menning et al., 2005). The authors used an auditory oddball design with frequent standard sentences to elicit a memory trace, which was interrupted by rare deviant (erroneous) sentences. Moreover, Hietanen and Nummenmaa (2011) revealed that N170 is sensitive to stimuli of naked bodies. In their studies it is even greater for nudes than to faces. Overall N170 seems to be an indicator for the preconscious individual importance of visual stimuli.

Finally the analysis of P300 component—an indicator for emotional operations—might reveal interesting results (Nieuwenhuis et al., 2005; Schupp et al., 2007; Flaisch et al., 2008). However, assuming that P300 is a correlate of conscious perception (Dehaene et al., 2006), more early EEG components like cited above should be considered first to capturing modes of more unconscious processes.

In sum these neurophysiological and the other cited attachment studies investigating implicit aspects of romantic attachment using self-report measures as explicit instruments for assessing attachment style (Marks and Vicary, 2015; De Carli et al., 2016) suggest that early visual and auditory stimuli could be used as a change detector of emotionally preferred stimuli. Thus, transposed to our tested and validated AAP RT paradigm, we would expect that the specific (secure or insecure) attachment system paves the way for a specific ERP, e.g., higher amplitudes or shorter latencies of the N170 or P300 to individual preferred stimuli which represent the own attachment representation. One advantage of our paradigm would be to use attachment related material linked to the individuals’ inner working models of attachment in a RT setting. This might extend previous studies in healthy samples and may provide some feasibility for clinical studies.

The measures based on RT reflect the overall activity of the brain needed for the elaboration of different stimuli. The results of the referred study suggested that the overall time needed for the processing of “unpleasant”, discomforting stimuli was higher than for “pleasant”, comforting ones. The fact that RT showed convergence with the individual’ inner working model of attachment in our study, has the potential to contribute to the validity of neurobiological experiments, like EEG. Therefore RT analysis with the proposed evaluation procedures might be of interest for a broader variety of questions concerning attachment in experimental and neurophysiological settings to capture automatic, unconscious processes in association with internal working models of attachment.

## Author Contributions

The study was conceptualized by AB, CG, TW and DP. The attachment experiment was developed by AB. The study setup and data collection were organized and conducted by TW. Coding of attachment interviews were conducted by AB. DP performed the statistical data analysis and contributed substantially to the result interpretation. DP developed the statistical procedure for RT analyses. CG, DP, TW, HM, IS and AB provided important intellectual contribution in commenting and revising the manuscript. AB, DP and TW wrote major parts of the manuscript and edited its final version.

## Funding

The publication is funded by the Faculty of Psychology and Sports Science, University of Innsbruck, Austria; Research Funding for Young Scientists.

## Conflict of Interest Statement

The authors declare that the research was conducted in the absence of any commercial or financial relationships that could be construed as a potential conflict of interest.

## Footnotes

^1^Technical note on ties: the ties were resolved by the replacing values by the average quantile value. For instance, by the subject p09, the first three quickest reactions needed the same time 0.185 s. Without ties, the ranks 1, 2 and 3 would correspond to the speed scores 76.6, 72.7 and 70.6. Because of ties, the mean value of three of these scores 73.3 was considered, rather than 72.2 corresponding to the mean rank 2.0. Resolving ties in this way, the intra-individual mean value was exactly 50.000 for each participant, the intra-individual standard deviations were very close to the value 10.000 (9.981–9.989), depending on the number of ties.

^2^An analogous approach is being used in the computer science: the complexity of a problem is classically operationalized by the number of needed steps of the problem solving algorithm, and consequently by the time demanded for the problem solution; see Sedgewick and Wayne (2011).

## Abbreviations

AAP: Adult Attachment Projective Picture System
AAP-RC: Adult Attachment Projective Relationship Choices Version 1
AAP-RC 2.0: Adult Attachment Projective Relationship Choices Version 2
ART: Attachment Reaction Times
Ds: dismissing attachment
E: preoccupied attachment
EEG: Electroencephalography
ERP: Event related potential
F: secure attachment
fMRI: Functional magnetic resonance imaging
RT: reaction times
U: unresolved trauma.
